# The role of progesterone and estrogen receptors in treatment choice after endometriosis surgery: A cross-sectional study

**DOI:** 10.18502/ijrm.v22i7.16970

**Published:** 2024-09-12

**Authors:** Tahereh Poordast, Saeed Alborzi, Ziba Kiani, Navid Omidifar, Elham Askary, Kefayat Chamanara, Mansoureh Shokripour, Alimohammad Keshtvarz Hesam Abadi

**Affiliations:** ^1^Department of Obstetrics and Gynecology, School of Medicine, Infertility Research Center, Shiraz University of Medical Sciences, Shiraz, Iran.; ^2^Department of Obstetrics and Gynecology, School of Medicine, Laparoscopy Research Center, Shiraz University of Medical Sciences, Shiraz, Iran.; ^3^Department of Obstetrics and Gynecology, School of Medicine, Maternal-Fetal Medicine Research Center, Shiraz University of Medical Sciences, Shiraz, Iran.; ^4^Department of Pathology, School of Medicine, Shiraz University of Medical Sciences, Shiraz, Iran.; ^5^Department of Biostatistics, Shiraz University of Medical Sciences, Shiraz, Iran.

**Keywords:** Endometriosis, Estrogen receptor, Progesterone receptor, Recurrence.

## Abstract

**Background:**

The lack of improvement in some endometriotic people's pain after surgery even while using hormone treatment may suggest an inappropriate response to routine hormonal therapies.

**Objective:**

This study aimed to determine a cut-off point for selecting the most appropriate treatment based on the hormone receptors of endometriotic lesions.

**Materials and Methods:**

In this cross-sectional study, by reviewing the medical records of participants and testing their archive samples and phone interviews (if needed), 86 symptomatic women after endometriosis surgery who were operated into governmental hospitals, Shahid Faghihi and Hazrate Zeinab Shiraz Iran were enrolled between March 2017 and March 2019. Women were divided into 2 groups: responsiveness (n = 73 for dysmenorrhea, n = 60 for dyspareunia) to medical treatment and surgery, and unresponsiveness (n = 13, n = 7). We examined the pathological slides of 86 women to determine the amount of hormone receptors and the relationship between the type of medical treatment and the level of hormone receptors on pain relief within 1 yr after surgery.

**Results:**

Based on the receiver operating characteristic curve, dysmenorrhea in the presence of tissue estrogen receptors > 60% (p = 0.1065), and dyspareunia in the presence of tissue progesterone receptors > 80% (p = 0.0001) responded well to medical treatment after surgery. In the presence of endometrioma-dysmenorrhea showed the best response to oral contraceptive pills (69.4%), while in deep infiltrative endometriosis-dyspareunia showed the best response to progesterone treatment (75%).

**Conclusion:**

Prescribing an appropriate hormone therapy based on a specific immunohistochemistry staining pattern can improve the life quality of postoperative endometriosis individuals.

## 1. Introduction

Endometriosis is a complex disease commonly seen in about 10% of women of reproductive age with presence of endometrial glands and stroma outside the uterine cavity (1). Symptoms like dysmenorrhea, dyschezia, dyspareunia, as well as infertility are associated with endometriosis, all of which can affect a person's quality of life significantly (2, 3).

The objective of endometriosis treatment is to reduce inflammation, disease activity, and alleviate the pain. For this purpose, several treatments have been suggested, including hormonal and nonhormonal therapies, (such as gonadotropin-releasing hormone, oral contraceptive pill [OCP], progestin, and nonsteroidal anti-inflammatory drugs) (4). Several hypotheses justify the onset and recurrence of endometriotic lesions; however, estrogen dependence and progesterone resistance have been shown to play a role in the recurrence of endometriotic lesions after surgery (5). In the endometrial implants, estradiol typically stimulates cell proliferation and progesterone stimulates cell differentiation. The function of ovarian hormones is mediated by estrogen (α and β) or (1 and 2) and progesterone receptors (PRs) (A and B). Previous studies have shown that the overexpression of hormone receptors, such as high estrogen receptor (ER)2/ER1 ratio and reduced PR expression play a role in endometriotic lesions. This receptor-mediated signal disorder affects cellular behavior and causes different responses to hormonal therapies (6–8).

One of the most common hormonal treatments for endometriosis is the use of progesterone or the compounds containing it. Progesterone (preg-4-ene-3, 20-dione) is a natural cholesterol catabolite of cyclopentanephydrofentanthrene (cyclopentanoperhydrophenanthrene) that is naturally produced in the corpus luteum (7). Progesterone functions by regulating endometrial decidualization and inhibiting estrogen-derived endometrial proliferation (7, 8). However, in some patients it has been observed that despite the similarity of serum progesterone levels in healthy and endometriotic women, endometriotic lesions do not respond adequately to progesterone (9). In endometriotic lesions, PR expression seems to undergo some changes (10–13).

On the other hand, available sources strongly support the benefits associated with the long term use of hormone therapy after surgery to prevent the recurrence of endometriosis and related symptoms, particularly dysmenorrhea. The lack of improvement in some patients may suggest an inappropriate response to the routine hormonal therapies in these cases. This is, especially true for those who relapse despite receiving hormone therapy after surgery (7, 10–15).

In addition, the production of estradiol (intracrine and paracrine) in endometriotic lesions increases the concentration of steroid hormones and enhances the estrogenic effect. Endometriotic lesions exert a lower level of estradiol inactivation compared to eutopic endometrium, which may further enhance the local effects. A few studies have investigated the role of PR in patients with endometriosis and its effect on treatment failure and disease recurrence (7, 13, 15–17). These studies have mainly focused on dysmenorrhea and endometrioma recurrence rather than dyspareunia and deep infiltrating endometriosis (DIE) lesions (16).

Therefore, the present research aimed to measure the levels of PR and ER in endometriotic lesions and determine a cut-off point for selecting the appropriate treatment based on the hormone receptors of these lesions to help improve the quality of life in patients with endometriosis.

The current article titled “Evaluation of progesterone and estrogen receptor (PR & ER) levels and their role in medical treatment of endometriosis patients" was published on Research Square's preprint site in October 2021 with DOI: 10.21203/rs.3.rs991753/v1.

## 2. Materials and Methods

In this cross-sectional study, from March 2017–2019, 1500 endometriotic women were referred to Shahid Faghihi and Hazrate Zeinab hospitals, Shiraz, Iran for endometriosis surgery based on the inclusion and exclusion criteria. By reviewing the medical records of patients and testing their archive samples and phone interview if needed, only 86 people were ultimately included in the study.

All women were divided into 2 groups: responsiveness to medical treatment and surgery, and unresponsiveness to surgery and medical treatment. Nonresponse criteria consisted of individuals whose endometriosis pain did not show improvement before the surgery, or experienced pain recurrence with a visual analogue scale (VAS) score > 5 during the 6- and 12-month follow-up visits. They showed no signs of recurrence in the subsequent ultrasound. All individuals were examined and analyzed separately regarding 2 common symptoms of endometriosis, that is, dysmenorrhea and dyspareunia. The relationship between the response to pain of each group was investigated and reported based on the amount of tissue hormone receptors as a result.

The inclusion criteria were: Women with a definitive diagnosis of endometriosis based on a pathology report, complete demographic, and follow-up information retrieved over a phone call (every 6 months, for at least 1 yr after the surgery), those whose VAS score in all the pain symptoms of endometriosis (dysmenorrhea or dyspareunia) were moderate to severe before the surgery (18), those with data about pain response to progesterone-based therapies after surgical treatment, unwillingness to conceive until at least 2 yr after the surgery, a tendency to continue the treatment, despite knowing that they will not have a menstruation period during this time, and women with a regular follow-up after the surgery, and no contraindication for hormonal treatment.

The exclusion criteria were: Incomplete records and follow-up, women with gastrointestinal or urinary tract diseases, or pelvic inflammatory disease, were on hormonal or infertility drugs up to 6 months before the surgery, had previous endometriosis surgery, follicle-stimulating hormone > 10 before the operation, age > 45 at the time of operation, subjects who for some reason stopped taking medications following the operation or used them irregularly, the existence of concomitant malignancy, and unavailability of pathology slides.

### Sample size

Out of 1500 women who underwent surgery, 96 of them met the inclusion criteria, whose medical records were analyzed to assess their response to medical treatment. 100% of them complained of dysmenorrhea and 80% complained of simultaneous preoperative dyspareunia. Meanwhile, in only 86 individuals, the tissue samples were sufficient for pathological examination.

### Data collection

Data were extracted from participants' medical records. Demographic information, such as age, body mass index, pain symptoms, and the stage of endometriosis disease according to the American Society for Reproductive Medicine classification, the affected area, the type of hormone therapies, treatment duration, and response to the treatment according to VAS were recorded in a checklist.

After all, adhesions were lysed and excised by sharp dissection to fully mobilize the ovaries and ovarian cystectomy; all the areas of superficial active endometriosis involving the other ovary or the pelvic peritoneum were fulgurated. Deep infiltrative endometriotic lesions located in the uterosacral, retrocervical, and rectovaginal area, Douglas pouch, rectum, and bladder were separated and resected from the surrounding normal tissue; while preserving important structures such as the ureter, uterine vessels, and pelvic nerves.

The participants were aware of the 2 treatment methods before their operation and were informed that none had been proven to be superior yet. All the participants were either at stage 3 or 4 of the disease according to the American Society for Reproductive Medicine classification. Medical treatment was initiated on the day of discharge based on the patient's preference, which included 30 mg daily medroxyprogesterone (Medrofem tablet 5 mg, Iran Hormone Co., Iran) or contraceptive pills with 0.03 mg ethinyl estradiol and 15 ug desogestrel (Desoceptive tablet, Iran Hormone Co., Iran). Medication was administered on a seasonal basis. The women were screened for the recurrence of the disease every 6 months (by ultrasound imaging). During the follow-up period, pain symptoms were checked and recorded at each visit. All the patients were followed-up for at least 12 months after the surgery (8–25 months). Failure in response to medical treatment was defined as new onset or persistence of endometriosis-related pain symptoms in the absence of recurrence of disease in 6 and 12 months of follow-up visits as a VAS score > 5. No recurrence was reported during the follow-up period.

After selecting the subjects based on the inclusion and exclusion criteria and extracting the required data from the patients' records, patients' surgical pathological slides were re-examined and stained for PR and ER for immunohistochemistry.

Primarily, the samples in paraffin were cut down to a size of 5 µm and placed on a slide (19, 20). The slides were deparaffinized and hydrated by a series of washes with xylene and ethanol. After rinsing in distilled water for 5 min, the slides were immersed in 0.01 M sodium citrate buffer for 15 min and then cooled down for 45 min.

The slides were then rinsed in phosphate-buffered saline (PBS) 1%, phosphate-buffered saline tween 20 (PBST) for 5 min (Thermo Scientific Pierce 20X PBS tween-20 was a space-saving stock solution ideal for preparing PBS-tween [PBS-T] wash buffers for the enzyme-linked immunosorbent assay, Western, and other immunoassays as well as a blocking buffer for plate-based assays) and cut with a hydrophobic pen. Endogenous peroxidase was quenched for 5 min with 3% hydrogen peroxide and then rinsed with PBST for 5 min. Nonspecific binding with 5% natural goat serum in PBST was blocked for 1 hr at room temperature.

The primarily used antibodies (PR H-190) (sc_7208; 1: 800) were purchased from Santa Cruz Biotechnology (Santa Cruz, CA). The slides were incubated with the initial antibody at 4 C overnight. Normal goat IgG (Biotechnology Santa Cruz, CA) was used as a negative control.

Natural endometrium on day 14 was also considered a positive control. Goat-antirabbit biotinylated-secondary antibody was utilized for PR (Vector Laboratories, Burlingame, CA) for 1 hr at room temperature. The slides were washed in 1% PBS and incubated at ABC Elite (Vector Laboratories) for 30 min at room temperature, then washed again with 1% PBS, and incubated with diaminobenzidine (Vector Laboratories) for 41 sec.

They were subsequently exposed to hematoxylin as a counterstain for 30 sec, and finally, rinsed with ethanol and xylene for 5 min and washed and mounted with permount (Thermo Fisher Scientific, Waltham, MA) (7, 21, 22). For each slide, the histopathologic score (H-score) for immunohistochemical staining was determined based on the receptor staining percentage. 2 pathologists separately scored each slide unaware of the patients, and the H-scores were average. The H-score was calculated using the modified version, expressed as negative (score 0), weakly positive (score 1), positive (score 2), and strongly positive (score 3) (8, 22, 23).

### Ethical considerations

The Ethics Committee of Shiraz University of Medical Sciences, Shiraz, Iran approved this research (Code: IR.SUMS.REC.1398.1395). All the procedures followed were in accordance with the ethical standards of the responsible committee on human experimentation (institutional and national) and with the Helsinki Declaration of 1964 and its later amendments. Informed consent was obtained from all the participants for using their medical information on the first visit after surgery. This was done so that their information files and histological samples archived in the pathology department at the first visit in both clinics could be used.

### Statistical analysis

The collected data were entered in Statistical Package for the Social Sciences, version 20.0, Statistical Package for the Social Sciences (SPSS) Inc., Chicago, Illinois, USA. For the final analysis, the participants were divided into 2 groups based on their response to the treatment, the data of the 2 groups were then compared. Qualitative data were compared using the Chi-square test and if necessary, Fisher's exact test, the quantitative data were compared using the *t *test. P < 0.05 were considered statistically significant.

## 3. Results

In this study, 96 women met the inclusion criteria, whose medical records were analyzed to assess their response to medical treatment. 100% of them complained of dysmenorrhea and 80% complained of simultaneous preoperative dyspareunia. Meanwhile, in only 86 individuals, the tissue samples were sufficient for pathological examination.

Demographic characteristics based on the improvement of dysmenorrhea or dyspareunia were the same for the responsive and nonresponsive groups (Table I). The women's mean age and mean body mass index were 34.71 ± 6.01 and 24.04 ± 4.16, respectively. Based on receiver operating characteristic (ROC) curve analysis with 2 threshold strategies, all individuals were categorized into 3 groups based on estrogen and PR density: low, moderate, and high receptor density as shown in the table II.

According to the data analysis, an H-score ≤ 5 was selected owing to high sensitivity (100%), and an H-score ≥ 80 for the PR and ≥ 70 for the ER were selected because of high specificity (100%).

Response to treatment of dysmenorrhea and dyspareunia (VAS < 3) was directly related to the increase of H-score, which is shown in table II. By elevating the PR and estrogen H-score from medium to high, the treatment response rises. While this enhanced response to treatment with increased tissue receptor was statistically significant solely in the high ER group (p < 0.05).

Due to a small sample size in the group with a low H-score, the results obtained from this group could not be considered valid.

**Figure 1:**
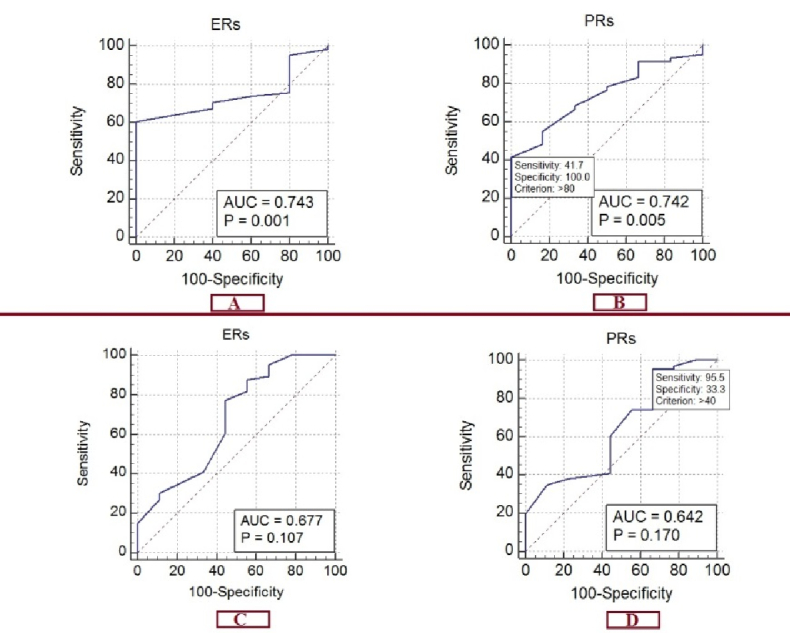
A, B) ROC curve for predicting the dysmenorrhea, C, D) and dyspareunia response to the treatment based on the H-score of PR and ER. AUC: Area under the curve, PR: Progesterone receptor, ER: Estrogen receptor.

As shown in figure 1, the ROC curve for predicting the dysmenorrhea response was based on the H-score, which shows an area below the curve of 0.677 for ER (95% CI: 0.559–0.780). This predicts a good response of dysmenorrhea to treatment with a sensitivity of 77.27% and a specificity of 55.56% in the presence of 60% ER in the tissue sample (p = 0.1065). For PR, the area below the curve was 0.642 (95% CI: 0.523–0.750), predicting the response to the treatment of dysmenorrhea with a sensitivity of 95.45% and a specificity of 33.33% in the presence of 40% PR in the tissue sample (p = 0.1699).

The ROC curve for predicting the dyspareunia response is based on the H-score for the ER, which shows an area below the curve of 0.743 (95% CI: 0.620–0.842). This predicts the response of dyspareunia to treatment with a sensitivity of 60.66% and a specificity of 100% in the presence of 70% ER in the tissue sample (p = 0.001). The area under the curve is 0.742 (95% CI: 0.619–0.842) for PR. This predicts the response of dyspareunia to the treatment with a sensitivity of 41.67% and a specificity of 100% in the presence of 80% of PR in the tissue sample (p = 0.005).

The rates of improvement for dysmenorrhea and dyspareunia are summarized in table III based on the cutoff presented in ROC curve for 3 types of endometriosis lesions.

**Figure 2:**
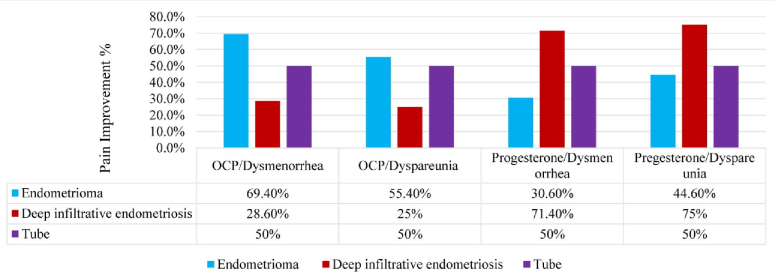
Endometriosis pain relief based on the lesion location and type of the treatment. OCP: Oral contraceptive pills.

Based on figure 2, while the type of treatment for tubal lesions does not show a difference in pain reduction, OCP treatment appears to yield a better pain response in endometrioma lesions, and progesterone treatment demonstrates a better pain response in DIE lesions, although neither of these differences are statistically significant (p > 0.05).

**Table 1:** Demographic characteristics and clinical data of patients with endometriosis


**Variable**		**Dysmenorrhea**			**Dyspareunia**		
		**Yes**	**No**	**P-value**	**Yes**	**No**	**P-value**
**Economic situation**							
	**Poor**	39 (48.8)	9 (56.3)		35 (52.2)	13 (44.8)	
	**Normal**	34 (42.5)	7 (43.7)		28 (41.8)	13 (44.8)	
	**Rich**	7 (8.8)	0	0.46*	4 (6)	3 (10.4)	0.67**
**Infertility**							
	**Yes**	9 (11.3)	2 (12.5)			8 (11.9)		3 (10.3)	
	**No**	71 (88.7)	14 (87.5)	> 0.99*	59 (88.1)	26 (89.7)	0.82*
**Menstrual cycle**							
	**Regular**	60 (75)	12 (75)		50 (74.6)	22 (75.9)	
	**Irregular**	20 (25)	4 (25)	> 0.99*	17 (25.4)	7 (24.1)	0.89*
**Type of treatment**							
	**GnRH-a**	1 (1.3)	0		1 (1.5)	0	
	**OCP**	41 (51.2)	8 (50)		29 (43.3)	20 (69)	
	**Progesterone**	38 (47.5)	8 (50)	0.89**	37 (55.2)	9 (31)	0.06**
Data are presented as n (%). * Chi-square test, ** Fisher's exact test. GnRH-a: Gonadotropin-releasing hormone agonist, OCP: Oral contraceptive pills							

**Table 2:** Response prediction based on dysmenorrhea and dyspareunia using PR and ER status


**H-SCORE**		**Dysmenorrhea**			**Dyspareunia**		
		**No**	**Yes**	**Response rate%**	**No**	**Yes**	**Response rate%**
**PR **							
	**High > 80**	3	41	93.2	5	39	88.6
	**Medium (6–80)**	8	31	79.5	7	32	82.1
	**Low ≤ 5**	0	3	-	0	3	-
**ER **							
	**High > 70**	3	45	93.8	2	46	95.8
	**Medium (6–70)**	8	29	78.4	7	30	81.1
	**Low ≤ 5**	0	1	-	0	1	-
Data presented as numbers. ER and PR: Estrogen and progesterone receptor							

**Table 3:** Endometriosis pain relief based on the lesion location and the level of hormone receptors


**Variables**	**Dysmenorrhea**				**Dyspareunia**			
**Receptor types**	**ER**		**PR**		**ER**		**PR**	
**Receptor cut-off**	> 60	< 60	> 40	< 40	> 70	< 70	> 80	< 80
**Endometrioma**	75.6	24.4	90.2	9.8	70.7	29.3	43.9	56.1
**Deep infiltrative endometriosis**	67.7	32.3	86.7	13.3	61.3	38.7	60	40
**Tube**	78.6	21.4	78.6	21.4	78.6	21.4	57.1	42.9
**P-value**	0.671		0.530		0.472		0.369	
Data presented as percentages, Chi-square test. ER and PR: Estrogen and progesterone receptors								

## 4. Discussion

This study showed that the levels of progesterone and ERs in endometriotic lesions could strongly predict the response of these patients to drug therapy (including OCP and progesterone). Our results revealed that the response to treatment of dysmenorrhea and dyspareunia is directly related to the increase in H-score. Based on previous studies in this field and those on breast lesions, we decided to set a threshold for the number of estrogen and PR in endometriotic lesions (17–24).

Therefore, we set a cut-off point to predict the response to the treatment of these lesions with the following goals: to improve the quality of life in these women and reduce the financial burden of this disease on society given the long-term need for treatment and follow-up. Accordingly, for predicting the appropriate response of dyspareunia to the treatment based on the ROC curve (p < 0.05), we set a 70% threshold for tissue estrogen and an 80% threshold for PRs, along with a 60% threshold for tissue estrogen and a 40% for PR (p > 0.05) to predict the appropriate response of dyspareunia and dysmenorrhea to the treatment.

Based on our study results, in the case of endometrioma (OMA), DIE, and tubal lesions pain symptoms responded better to the treatment once the estrogen and PR levels were higher than the cut-off point. Comparing the pain symptoms improvement and the role of determining tissue hormone receptors, it should be said that in most OMA lesions, tissue ERs were determined more than the cut-off point. While in the DIE lesions, tissue PR were determined more than the cut-off point, which justifies the response to the treatment of lesions to OCP or progesterone treatment; however, this difference was not statistically significant.

A few studies have investigated the status of PR and ER and its role in predicting the treatment response in patients with endometriosis (21, 23–28). Similarly, Flores et al., 2018 examined the status of PR and its role in predicting the response of endometriotic lesions to progesterone treatment. In their study, H-score was used to determine the qualitative status of PR. H-score was higher in responsive patients, and the treatment response status was strongly associated with PR. They concluded that depending on the number of receptors in endometriosis, different hormone-based therapies could be pursued after surgery (15).

In 2017, Hou et al., examined the role of predictive biomarkers in the accurate treatment of endometriosis. They investigated the effect of bazedoxifene and medroxyprogesterone acetate on the expression of PR, ER, and aromatase enzyme (*CYP19A1*) genes in the cell culture media obtained from the patient biopsy with endometriosis. They concluded that the degree of PR expression might predict progesterone resistance as well as response to treatment of endometriotic lesions (29).

It is yet to be determined, whether the heterogeneity observed in the expression of hormone receptors in different types of tissues can explain the difference in patients' response to hormone therapy (30). Based on table III, concerning OMA, DIE, and tubal lesions dysmenorrhea, and especially dyspareunia, responded better to the treatment if estrogen and PR levels were higher than the cut-off point. Our obtained data also confirmed previous findings that showed variable levels of PR in various endometriotic lesions. Thus, it is best to treat all endometriotic lesions based on the expression of their hormone receptors (31).

In line with the present study, Colón-Caraballo et al., 2019 conducted their research aiming at expressing the concentrations of steroid receptor hormones in different types of endometriotic lesions and also eutopic endometrium. This comparison was performed between the endometriosis and normal women (control group) using the tissue microarray method. Their results indicated that ovarian lesions had the lowest expression of ER1 (alpha estrogen) and PGR (progesterone) and the highest ER2 (beta estrogen) expression, while the highest expression of all 3 receptors belonged to the fallopian tube lesions. The highest ER2: ER1 ratio was observed in ovarian and endometrial secretory lesions (32). Other studies in this area also show overexpression of ER2/ERS1 in endometriosis tissues, which leads to increased proliferation in lesions and also induces progesterone resistance (13, 14, 33, 34).

This shows a clear association between ER and PR expression, and its secretion cycle with the development and progression of endometriosis. Increased expression of B ER leads to more local production of estrogen as well as suppression of PR in endometriosis tissue. In addition, a lack of PR in these tissues was observed, which leads to progesterone resistance, commonly reported as estrogen-dependent and progesterone-resistant in the endometriosis tissue (32). Thus, the IHC expression characteristics of nuclear isoforms of ER and PR in target tissue sampled during surgery can predict the response to commonly prescribed drugs.

Given the fact that the overexpression of ER2/ER1 in hormone-dependent malignancies, such as breast and prostate cancer, and also endometrial cancers, are associated with a high-grade tumoral process and predicts clinical outcomes, including the overall survival rate of patients, we can use the results of this study for the same purposes in patients with endometriosis (13, 35–37). In our study, the improvement of dysmenorrhea and dyspareunia in tubular lesions did not depend on the type of treatment, but in the case of the OMA, dysmenorrhea responded better to the treatment with OCP. Additionally, in the case of DIE lesions, the dysmenorrhea and dyspareunia responded better to the treatment with progesterone compared with those in OCP. Hence, we found a difference in PR levels between the patients and even in an individual, based on the type of endometriotic lesions which can predict the treatment response in endometriotic people and help to choose a better treatment for each individual. It may also prevent the recurrence of the disease following surgery.

The significant improvement in dyspareunia compared to dysmenorrhea in our study confirmed the higher PR levels of pelvic endometriotic lesions compared with ovarian endometrioma lesions (13).

According to our findings, a gynecologist can opt for the right hormonal treatment (such as OCP and progesterone) based on the specific pattern of IHC staining obtained from the patients surgical specimens, resulting in improved quality of life and effective pain reduction. Therefore, disease recurrence in the reproductive age may be prevented by prescribing an appropriate treatment. The limitation of our study is the lack of estrogen subtype determination, since through comparing the difference in ER2/1 ratio in endometrioma, other DIE lesions, and the short length and follow-up time for evaluation of the recurrence rate based on the type of treatment and concentration of hormone receptor in these lesions.

## 5. Conclusion

Prescribing the correct hormone therapy based on a specific IHC staining pattern can enhance the quality of life for postoperative endometriosis patients and decrease the likelihood of pain recurrence. Therefore, gynecologists can recommend appropriate hormonal treatments (like OCP and progesterone) by analyzing the specific pattern of IHC staining from patients' surgical samples, resulting in improved quality of life and effective pain relief postsurgery.

##  Data availability

Study data can be shared electronically through the corresponding author's email if necessary.

##  Author contributions

Tahereh Poordast: Conception and design of study, Saeed Alborzi: Conception and design of study, Navid Omidfar: Conception, design of study, and patient recruitment, Kefayat Chamanara: Patient recruitment, and manuscript preparation, Mansoureh Shokripour: Conception and design of study, and patient recruitment. Ziba Kiani: Patient recruitment & data collection. Elham Askary: Conception, design of study, final approach, data interpretation, and manuscript preparation. Alimohammad Keshtvarz Hesam Abadi: Data analysis, interpretation, and final approach.

##  Conflict of Interest

The authors declare that there is no conflict of interest.
